# Evolution of Antiviral Drug Resistance in SARS-CoV-2

**DOI:** 10.3390/v17050722

**Published:** 2025-05-18

**Authors:** Roy Dinata, Piyush Baindara, Santi M. Mandal

**Affiliations:** 1 Animal Science Research Center, Division of Animal Sciences, University of Missouri, Columbia, MO 65211, USA; dinataroy9@gmail.com; 2 Department of Chemistry and Biochemistry, University of California San Diego, La Jolla, CA 92093, USA; mandalsm@gmail.com

**Keywords:** SARS-CoV-2, COVID-19, mutations, drug resistance, antivirals

## Abstract

The COVID-19 pandemic has had a significant impact and continues to alarm the entire world due to the rapid emergence of new variants, even after mass vaccinations. There is still an urgent need for new antivirals or strategies to combat the SARS-CoV-2 infections; however, we have success stories with nirmatrelvir. Drug repurposing and drug discovery may lead to a successful SARS-CoV-2 antiviral; however, rapid drug use may cause unexpected mutations and antiviral drug resistance. Conversely, novel variants of the SARS-CoV-2 can diminish the neutralizing efficacy of vaccines, thereby enhancing viral fitness and increasing the likelihood of drug resistance emergence. Additionally, the disposal of antivirals in wastewater also contributes to drug resistance. Overall, the present review summarizes the strategies and mechanisms involved in the development of drug resistance in SARS-CoV-2. Understanding the mechanism of antiviral resistance is crucial to mitigate the significant healthcare threat and to develop effective therapeutics against drug resistance.

## 1. Introduction

SARS-CoV-2 infection in respiratory airways causes acute respiratory distress syndrome (ARDS), which is the major disease symptom accompanying morbidity and mortality; however, there are other multiple disease symptoms associated with COVID-19 along with secondary bacterial and fungal infections [1,2,3,4]. Remdesivir and nirmatrelvir are the only FDA-approved antivirals, authorized in October 2020 and May 2023, respectively, while molnupiravir is permitted solely for emergency usage (Table 1). Additionally, several vaccines are available (Available online: https://www.cdc.gov/covid/vaccines/index.html (22 April 2025)); however, the rapid emergence of new mutants has always remained a challenge. In the absence of efficient and approved therapeutic strategies against new variants of SARS-CoV-2, several efforts have been made in the recent past. As a consequence, the use, testing, and repurposing of multiple drugs developed a selective pressure on the virus that may subsequently result in emergence of antiviral drug resistance. The development of antiviral drug resistance provides important information about the novel antiviral mechanisms of the drugs and their efficacy; however, properly regulated surveillance for the emergence of resistance is essential for public health and our healthcare system throughout the COVID-19 pandemic.

RNA-dependent RNA polymerase (RdRp) of SARS-CoV-2 is the prime interest for drug development due to its high conservation of amino acids within the beta-coronaviridae virus family and is so profoundly targeted for drug repurposing [5]. Several RdRp inhibitors including remdesivir, sofosbuvir, galidesivir, tenofovir, and ribavirin have been tested for their efficacy against SARS-CoV-2; however, the first available antiviral treatments were 3C-like protease inhibitors such as lopinavir/ritonavir [6,7,8]. While only remdesivir has been approved by WHO as an antiviral drug to treat COVID-19, all the RdRp inhibitors further develop a selective pressure on the virus that may contribute to the emergence of drug resistance as they all are nucleoside analogs and have a common mechanism of action.

Proving the fact of the emergence of drug resistance under selective pressure, a recent in vitro study using SARS-CoV (the nearest relative to SARS-CoV-2) that also causes ARDS found that definite single nucleotide polymorphisms (SNPs) in nsp12 may change its susceptibility to remdesivir [9]. Furthermore, SARS-CoV cultures having Nsp12:Phe480Leu or Nsp12:Val557Leu mutations were found six times less susceptible to remdesivir while in the absence of remdesivir reduced viral fitness and inefficient replication were observed in these mutants [10]. Additionally, mutational analysis in SARS-CoV-2 main protease (Mpro) variants in the presence of nirmatrelvir revealed E166V, L27V, N142S, A173V, and Y154N and their combination as the most common mutations involved in resistance to nirmatrelvir. However, the interpretation of the results suggested a slower resistance development rate against nirmatrelvir [11]. Overall, increasing use or testing of antiviral drugs and long-term treatments with similar antivirals could be a major reason behind the increased rate of viral mutations and the subsequent emergence of resistance towards antiviral drugs. Furthermore, resistance incidences can be controlled by different control measures and rational use of antiviral drugs. The combination of multiple drugs with different mechanisms of action could be another strategy to limit the emergence of drug resistance. In total, identification and close surveillance of antiviral or highly mutated viral strains are necessary to keep track of the emergence of antiviral drug resistance. In the present study, we summarized the major antiviral drug-resistance development mechanisms in different SARS-CoV-2 variants.

## 2. Current Status of Antiviral Resistance in SARS-CoV-2

Antiviral drug-resistance development is a complex process that is affected by the host as well as viral factors. Antiviral drug potency and genetic barriers are prominent keys behind drug-resistance development along viral pathogenicity and overall fitness. First of all, RNA viruses lack the high-fidelity proofreading machinery and are thus known to have high genetic variability [12]. This leads to high mutation rates along with low replication fidelity, while on the other hand coronaviruses including SARS-CoV-2 are an exception to this and have exoribonuclease proofreading activity in the nonstructural protein 14 (ExoN) [13]. Interestingly, proofreading activity in coronaviruses makes them less prone to acquire high mutations but at the same time makes them able to escape nucleotide analogs antiviral drugs [9]. Notably, SARS-CoV-2 susceptibility towards remdesivir, a most widely used class of broad-spectrum antiviral drugs, is also regulated by viral polymerase and proofreading exoribonuclease [10]. Resistance to remdesivir has been observed in murine coronavirus after several passages; however, this is not the truth for SARS-CoV-2. SARS-CoV-2 showed resistance against remdesivir in less than a year when it was approved by the FDA for SARS-CoV-2 infection [10,14]. On the other hand, molnupiravir was found less prone to resistance development in murine coronavirus and MERS-CoV; however, few resistance reports are available against 3CL pro-inhibitors such as GRL-001 [15,16]. Recently, molnupiravir was found effective against SARS-CoV-2 infection by inhibiting its replication cycle, and human clinical trials are in progress currently [17,18]. A large clinical trial on the oral treatment of COVID-19 using molnupiravir in non-hospitalized patients concluded the reduced risk of hospitalization, and death in unvaccinated adults [19]. Although molnupiravir was found effective against SARS-CoV-2, there is still time for its availability in clinical practice for COVID-19 and there is always a chance to develop resistance against it, too [20]. There are several other antiviral or repurposed drugs suggested for the SARS-CoV-2 treatment but the majority are in the premature stage of clinical development, so limited data are available about antiviral resistance development in SARS-CoV-2 [21]. However, some comprehensive databases maintained the updated details as far as possible (https://covdb.stanford.edu). The fate of SARS-CoV-2 resistance development is unknown, but knowing antiviral drug-resistance development is crucial to developing effective pandemic treatments.

## 3. SARS-CoV-2 Variants: A Brief Window of Time and High Chances for Antiviral Drug Resistance

SARS-CoV-2 variants continue to evolve and roam throughout the world currently. Five new variants have been identified during the year 2021 and classified under the list of variants of concern by WHO. New variants are carrying several mutations that increase viral fitness and also make a virus spread quickly throughout the world. All the characteristics of new variants pose a high threat to the emergence of antiviral drug resistance in SARS-CoV-2 and require continuous surveillance using testing, screening, and sequencing of viral isolates from the newly infected patients. Specifically, most of the new mutations found in SARS-CoV-2 spike protein are related to the set of recurrent mutations [22].

SARS-CoV-2 variants are characterized by transmissibility, disease severity, and their ability to escape humoral immunity. Due to increased transmissibility, new variants outcompete other existing variants and quickly spread in the population while showing a higher reproduction rate and chances of secondary attack frequency compared to the older variants [23,24,25]. One of the deadliest SARS-CoV-2 variants omicron has only 6 unique mutations out of all 61 mutations while the rest already exist in the already sequenced genomic sequences of SARS-CoV-2. However, results showed that omicron could potentially escape the two doses of vaccines with a 33- to 44-fold reduction in antibody neutralization capabilities [26]. Interestingly, omicron has three sets of unique mutations that contribute to its increased transmissibility by different mechanisms. The spike protein mutations Q498R and N501Y contribute to increased binding with its receptor ACE2 [27]. Next, H655Y, N679K, and P681H triple mutations were found to play a role in improved cleavage at the S1/S2 junction while N protein mutations R203K and G204R participate in increased replication that boosts the viral load in patients [28]. The rapid evolution of SARS-CoV-2 variants in a short window of time with increased viral fitness poses a high risk towards the emergence of drug-resistance development; however, the long-term impact of these mutations on antiviral drug-resistance development is undetermined and needs further in-depth studies.

## 4. Role Players in the Drug-Resistance Development in SARS-CoV-2

**Figure 1:**
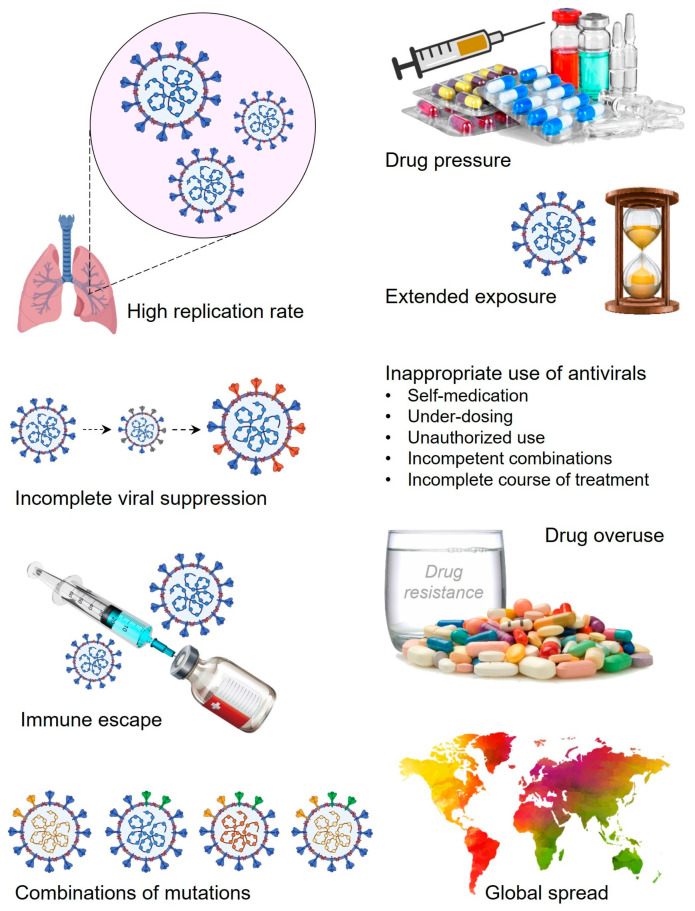
Different factors and role players in the antiviral drug resistance development in SARS-CoV-2.

High replication rate, multiple mutations, immune escape strategies, incomplete suppression, drug pressure, and prolonged exposure due to overuse, along with global spread, are the major causes behind the rapid emergence of drug resistance in SARS-CoV-2 [29]. The high replication rate of SARS-CoV-2 also allows a huge chance for the incorporation of mutations and errors in the genome. The accumulation of different mutations develops a high chance for the rapid emergence of antiviral drug resistance. Importantly, antiviral drugs generate a selective pressure that provides chances for rapid evolution of SARS-CoV-2 mutants [30]. Notably, selective SARS-CoV-2 mutants evolved under this selection pressure and survived well leading to antiviral drug resistance. Similarly, prolonged exposure to antiviral drugs allows better opportunities for SARS-CoV-2 mutants to overcome the antiviral drug-associated selective pressure in the form of continuous viral replication and evolution of drug resistance. On the other hand, incomplete viral suppression provides more opportunities for survival, evolution, and development of new resistance mechanisms against existing antivirals of SARS-CoV-2 [31]. Furthermore, infection with different mutants of SARS-CoV-2 at a single time point might exhibit a more complex situation for targeted antiviral drugs that also fail multiple drug combinations and even the evolution of new mutants of SARS-CoV-2 [32]. Additionally, injudicious use of antivirals including over- or under-dosing, self- and non-regulated medication, short-time or non-recommended courses, along with incompetent uses, potentially allows the development of antiviral drug resistance in SARS-CoV-2 [33]. Above all, the global spread and transmission of SARS-CoV-2 via different means significantly poses a great impact on the rapid evolution of drug resistance in SARS-CoV-2 that results in varied antiviral efficacies due to complex drug-resistant patterns. In summary, high replication rate, drug pressure, extended exposure to the antivirals, incomplete viral suppression, immune escape, overuse of antivirals, global spread, and injudicious use of antivirals such as self-medication, under-dosing, unauthorized use, and insufficient treatment periods are major factors behind the rapid emergence of drug resistance in SARS-CoV-2 (Figure 1).

### 4.1. Glycans-Associated Drug-Resistant Mechanism in SARS-CoV-2

**Figure 2:**
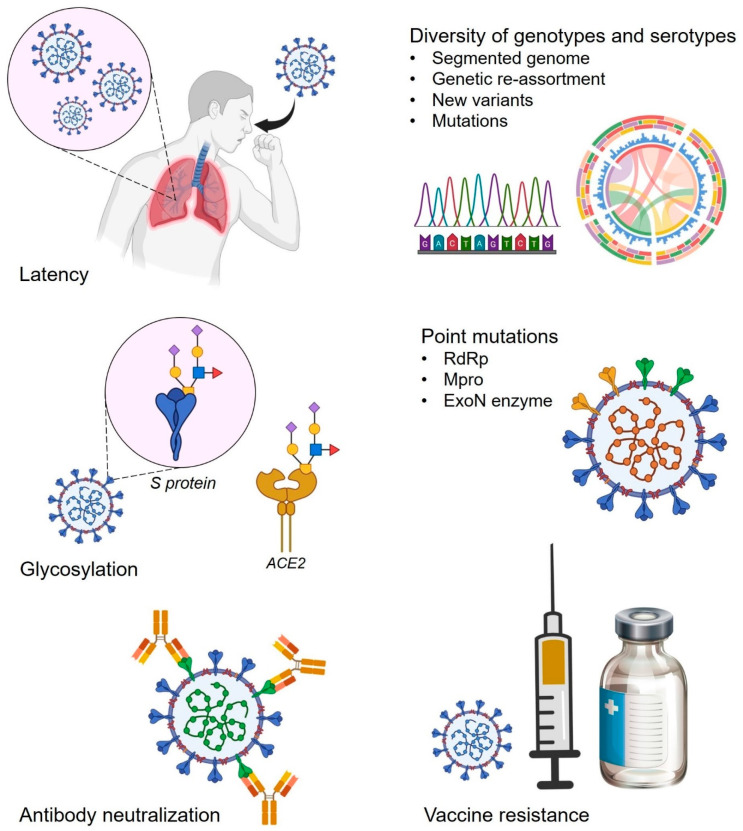
Major factors and antiviral drug resistance development mechanisms in SARS-CoV-2.

The spike protein of SARS-CoV-2 and its mammalian receptor ACE2 are both highly glycosylated. Notably, these glycans are essentially involved in and responsible for viral infectivity as well as in host immune dodging, thus playing an important role in the development of drug resistance [29]. Importantly, spike protein glycans alter their binding affinity towards ACE2 by modulating the conformation and stability that directly affects viral infectivity leading to resistance development. Similarly, glycans on the ACE2 such as N90 and N322 were demonstrated to play an essential role in spike protein binding. However, N90 glycosylation does interfere with binding and inhibit the viral docking on human cells while N322 glycosylation positively interacts with spike protein and stabilizes the viral–host interaction [34]. Additionally, mannosidase-I, a glycosylation inhibitor, has already been shown to restrict viral entry via modulating the glycosylation pattern of the SARS-CoV-2 spike protein [35]. Furthermore, glycosylation is also involved in and restricts immune recognition, vaccine efficiency, and dodging neutralizing antibodies [36]. Overall, glycans have been suggested to play an important role in drug-resistant development in SARS-CoV-2 and can be utilized as a potential tool in therapeutic development in the battle against the rapid emergence of drug-resistance development in SARS-CoV-2 (Figure 2).

### 4.2. Point Mutations and Associated Threat of Antiviral Resistance in SARS-CoV-2

The process of resistance development is mainly the acquired resistance among all infectious agents including bacteria, fungi, and viruses. Genetic polymorphism or mutations are the primary mechanism in resistance development which includes single nucleotide polymorphism (SNPs), insertion, and deletions. Point mutations are well documented for antibiotic resistance in bacteria; however, much less is reported for the role of point mutations during resistance development in viruses (Figure 2). We know that viruses can mutate at high frequencies whereas RNA viruses have 100-fold higher mutation rates in comparison to DNA viruses [37]. The fact is that the mutation rates are critically determining the chances of a mutation to be advising drug resistance, antibody escape, or vaccine resistance. RdRp in the replication machinery of RNA viruses, is the reason behind their high mutation frequencies. RdRp usually does not have proofreading activity and is unable to correct the replication mistakes. However, coronaviruses including SARS-CoV-2 are exceptions and have an RdRp-independent proofreading activity, thus seeming to have lower mutation rates than other RNA viruses [38]. However, there are at least 11 novel variants that have been reported to date around the world having various mutations in spike, NSP12, ORF3a, NSP2, NSP13, ORF8, and nucleocapsid protein, along with the 5′ UTR region of SARS-CoV-2 [39]. Notably, various upstream pathways are reported that are involved in the regulation of SARS-CoV-2 mutational defects while contributing to antiviral drug-resistance development. Entropic expansion of mutations involving intrinsically disordered regions of the SARS-CoV-2 proteome is an example of one such pathway. Viroporin protein 3a and N protein-involving pathways are reported to have a high impact that leads to mutational changes and thus antiviral resistance [40]. Interestingly, different variants or mutations may induce different symptoms in different individuals, while the factors causing this high frequency of mutations and their associated health consequences are not well understood. One of the major impacts of high mutation rates is the development of drug resistance which is not yet more of a concern and only a few studies are available regarding drug resistance development in SARS-CoV-2.

#### 4.2.1. Mutations in nsp12 Leading to Antiviral Resistance in SARS-CoV-2

Nsp12 is an essential part of the SARS-CoV-2 RNA polymerase involved in the RNA synthesis initiation of genetic replication. Nsp12 forms a functional RdRp complex by interacting with nsp7 and nsp8, which regulate the replication–transcription complex. Due to its crucial role in SARS-CoV-2 replication, nsp12 has been recognized and targeted as the main target for antivirals. Importantly, mutations in the active site of nsp12 could play a significant role in SARS-CoV-2 resistance development against antivirals. A recent in vitro study showed that only serial passaging of SARS-CoV-2 in the presence of remdesivir confers the drug-resistant viral population. In Vero cells, actively replicating SARS-CoV-2 strains in the presence of remdesivir showed at least a 2-fold increase in the IC50 values. While there was no change in the IC50 values in the absence of remdesivir, this suggested the development of a remdesivir-specific resistant mutant in vitro. It has been determined that a single mutation in RdRp, E802D, was sufficient to reduce the SARS-CoV-2 sensitivity against remdesivir. It is suggested that E802D mutation results in minor structural changes in RdRp at the binding site of remdesivir which reduces steric hindrance and affects the binding of nucleotides during the synthesis of template RNA and allows elongation even when the active form of remdesivir is incorporated into the RNA [14]. Notably, a similar mutation has been observed in an immunocompromised patient with the SARS-CoV-2 infection [41]. Next, a mutation in RdRp, Nsp12:F480L substitution, also creates steric hindrance and affects the remdesivir binding by destabilizing the interface between different domains of the RdRp [10]. Surprisingly, results suggested that spike protein mutants similar to recently emerging variants can arise in vitro, in the absence of immune pressure [14]. However, point mutations about the antiviral resistance mechanisms in SARS-CoV-2 are still not fully understood; RdRp (nsp12, nsp7, nsp8) of SARS-CoV-2 is significantly targeted by several inhibitors like remdesivir or molnupiravir [42,43]. Another recent study analyzed more than 56,000 SARS-CoV-2 genomes and confirmed selective negative pressure associated with nsp12 which might be a potential hot spot for antiviral escape mutations such as Nsp12:Val473 and Nsp12:Arg555 [44]. Also, genetic analysis identified two mutations, S759A and V792I, to confer resistance against GS-441524 (parent nucleoside of remdesivir) upon several passages [45]. In another case study, an intestinal pneumonia patient infected with Omicron BA.5 was found to have the genetic mutation C799F in nsp12 upon whole genome sequencing [46]. Overall, these mutations might destabilize the ligands of RdRp-targeted drugs including remdesivir and molnupiravir which possess a similar mechanism of action to inhibit RdRp (Table 2).

#### 4.2.2. Mutations in nsp5 and Antiviral Drug Resistance in SARS-CoV-2

SARS-CoV-2 protease (nsp5 or 3CLpro) or Mpro is essential for viral replication and gene expression while its structure along with functions are highly conserved. Mpro has already been targeted as the potential drug target to combat SARS-CoV-2; however, the rapid emergence of mutations within the drug-binding pocket of Mpro diminishes the efficacy of antiviral drugs such as nirmatrelvir and ensitrelvir. Screening of naturally occurring Mpro mutants revealed 22 mutations in five residues of the nirmatrelvir-binding pocket within Mpro (S144M/F/A/G/Y, M165T, E166 V/G/A, H172Q/F, and Q192T/S/L/A/I/P/H/V/W/C/F). Interestingly, all these mutants demonstrate similar enzymatic activity to the wild type; however, they exhibit significant resistance to nirmatrelvir [49]. Another study demonstrates multiple similar mutational pathways for resistance to nirmatrelvir, including T21I, P252L, or T304I as major precursor mutations. Out of all mutations, E166V is responsible for the highest resistance development, however, resulted in poor replicative fitness of SARS-CoV-2. Additionally, the L167F mutation is involved in steric clash with nirmatrelvir while the F140L mutation restricts the interaction of both nirmatrelvir and ensitrelvir with Mpro by destabilizing the π–π stacking interactions between F140 and H163 [53]. Cell culture studies also showed serial passage-induced resistance development in SARS-CoV-2 via substitutions such as L50F and E166V that restrict the nirmatrelvir binding to Mpro. Notably, L50F, E166V, and L50F + E166V confer reduced replication and Mpro activity while L50F and L50F + E166V variants showed high fitness. Interestingly, naturally occurring L50F mutation compensates for the fitness cost of E166V that induces viral escape and encourages the selection of nirmatrelvir-resistant mutants, thus expediting the resistance development [54]. Another study focusing on naturally occurring mutants of Mpro in SARS-CoV-2 identified A173V and M49L mutations as conferring the highest resistance to nirmatrelvir, and ensitrelvir, respectively [55,56]. Next, the global prevalence of Mpro mutations associated with nirmatrelvir and ensitrelvir has been analyzed among 13.4 million sequences submitted to GISAID and revealed G15S and T21I as the most widespread mutations [57]. In agreement with this, a recent clinical report demonstrates independent acquisition of T21I mutation in SARS-CoV-2 Mpro post nirmatrelvir exposure [58]. Similarly, a recent case study of an immunocompromised patient revealed de novo mutation, E166V, and L50F in Mpro after repeated and long exposure to nirmatrelvir which results in enhanced viral replication and treatment failure [50] (Table 2). Another thought-provoking study revealed the contribution of Mpro hyperactive mutation in the evolution of SARS-CoV-2 resistant development over time. These hyperactive mutations are discovered at both proximal and distal locations from the active site and play an essential role in functional dynamics that enhance the overall activity of Mpro. Interestingly, the abundance of hyperactive mutations is three times more than the nirmatrelvir binding mutation in Mpro, suggesting their possible role in SARS-CoV-2 drug-resistance evolution [59].

#### 4.2.3. ExoN Enzyme Promotes Antiviral Resistance in SARS-CoV-2

The transcription complex is the favorite target for antivirals against SARS-CoV-2 which also includes its core of RdRp and other non-structural proteins (NSPs). A recent study by Liu et. al. demonstrates the high proofreading activity of the ExoN enzyme that could correct the wrong inserts of nucleotide analogs like remdesivir more efficiently and resume the arrested replication of SARS-CoV-2 [60]. The ExoN enzyme is located at the N-terminal ExoN domain of the SARS-CoV-2, nsp14, and stimulated by nsp10 while the NSPs also contribute to the stabilization of its active site structure. Other than efficient proofreading, the ExoN enzyme also inhibits the host–pathogen recognition receptors (PRRs) by breaking down the SARS-CoV-2 double-stranded RNA (dsRNA). It has been discovered that a free 3′-OH of the RNA substrate is crucial for the ExoN enzyme-mediated breakdown of the dsRNA. This observation suggested that 3′-deoxy nucleotide analogs can effectively function as efficient RdRp chain terminators to arrest viral replication but at the same time are resistant to ExoN excision. These functional properties of the ExoN enzyme seem to better allow SARS-CoV-2’s immune escape capabilities that provide a chance to develop antiviral drug resistance over time, specifically against nucleoside analog antiviral drugs like remdesivir and molnupiravir [60].

### 4.3. Antibody Neutralization, Vaccine Resistance, and Associated Selection Pressure for Antiviral Drug Resistance in SARS-CoV-2

Currently, there are hundreds of vaccines under development for COVID-19 and several under clinical trials [61]. However, vaccine resistance may occur more often than antiviral drug resistance [62]. There are different reasons behind the development of vaccine resistance including antigenic drift, serotype replacement, and increased disease severity [63]. Extensive mutations in the different variants of SARS-CoV-2 may lead to antigenic changes that result in viral resistance against monoclonal antibody therapies. SARS-CoV-2 variants B.1.1.7 and B.1.351 are of specific concern as recently studied for their ability to escape from antibody neutralization that leads to antibody resistance [64]. Both of these variants were reported for their increased transmissibility due to several mutations in the spike gene; however, possible antigenic changes towards antibody neutralization significantly affect their sensitivity and could potentially contribute to the development of resistance. In a recent study, both variants, B.1.1.7 and B.1.351, were found to have resistance to neutralization by N-terminal domain (NTD) supersite-targeted monoclonal antibodies. Surprisingly, resistance is also found against a major subset of strong monoclonal antibodies that target ACE-2 binding sites (RBM, receptor-binding motif) [30]. However, convalescent plasma from the earlier infected patients from SARS-CoV-2 did not show a substantial change in neutralizing activity against B.1.1.7 but a significant reduction is observed against B.1.351 [65,66]. These observations suggested a possible development of resistance against antibody neutralization that is a major issue to consider and only the future can tell us the consequences. Recent studies from the Novavax vaccine trial in South Africa suggested the same as the patients who got the placebo with earlier SARS-CoV-2 infections showed sensitivity towards successive exposure to B.1.351 [67,68]. Another example is from Manaus, Brazil, where 76% of the population was seropositive because of earlier infection; even then, the second wave of infection caused by variant P.1 was spread widely [69]. It has been observed that activity loss of convalescent sera and vaccine-elicited antibodies against B.1.351 is higher than the results reported using mutant pseudoviruses [70,71]. Notably, convalescent plasma has recently been approved by the FDA in December 2024 for the treatment of COVID-19 in patients with immunocompromise-immunosuppressive diseases or receiving immunosuppressive treatments (Table 1). Overall, the situation is alarming, especially when the recent reports suggested a significant drop in the efficacy of Novavax and Johnson & Johnson vaccines in South Africa [67]. This is just the beginning of antigenic drift in SARS-CoV-2 that resulted in variants like B.1.1.7, B.1.351, and P.1. Different studies have confirmed that spike protein mutations accumulated in these variants are evolved under antibody selection pressure in vivo, and result in antibody neutralization leading to resistance [72,73].

### 4.4. Antiviral Drug Disposal in the Environment and Associated Risk of Antiviral Drug Resistance in SARS-CoV-2

It has already been reported that continuous leaks of antiviral drugs into the environment, especially in the aquatic environment or wastewater treatment plants (WWTPs), result in the development of antiviral drug resistance [74]. As the COVID-19 pandemic goes on, a continuous search for an effective antiviral drug for SARS-CoV-2 is ongoing worldwide. Several antiviral and antiparasitic drugs such as remdesivir, favipiravir, oseltamivir, lopinavir/ritonavir, and chloroquine have been tested, repurposed, or undergone clinical trials for COVID-19 patients [75,76]. The fact is that all these drugs along with their metabolites are excreted from the body, especially in the urine, after a certain time. Excretion of antiviral drugs from the body creates a potential chance of environmental leakage relying on the drug removal efficacy of WWTPs [77,78]. Treatment of contaminated wastewater at WWTPs results in residues and the formation of metabolites along with oxidation and transformation products, rather than complete mineralization. There is a chance for these residual compounds or byproducts to be active as their parental compounds, which is the major concern for the antiviral drug-resistance development via natural reservoir animals of the viruses. These natural reservoirs of the viruses such as bats, cats, pigs, camels, and pangolins have a potential chance to be exposed to water sources such as rivers that carry environmentally leaked antiviral drugs [79]. This indirect exposure of viral reservoirs to the antiviral drugs develops a selective pressure and mutations over time that lead to antiviral drug resistance [80]. There are high chances of acceleration in antiviral drug resistance upon exposure of animal reservoirs to the excreted antiviral drugs present in the wastewater. Likewise, anti-influenza drug resistance in the body of waterfowl (natural reservoir of influenza virus) has already been reported [81].

In the recent past, several studies reported the presence of SARS-CoV-2 and other micropollutants in hospital wastewater and wastewater plants [82,83]. These events of surface water exposure to SARS-CoV-2 and antiviral drugs during the pandemic suggested that SARS-CoV-2 likely has higher chances of acquiring antiviral drug resistance in animal reservoirs like bats and pangolins [84]. Environmental leakage of antiviral drugs and the development of drug resistance is a process happening over time that is already going on in the case of SARS-CoV-2. However, the long-term implications of antiviral drug resistance resulting from the events of the pandemic remain to be determined.

## 5. Management and Control Measures Against Antiviral Drug Resistance

**Figure 3:**
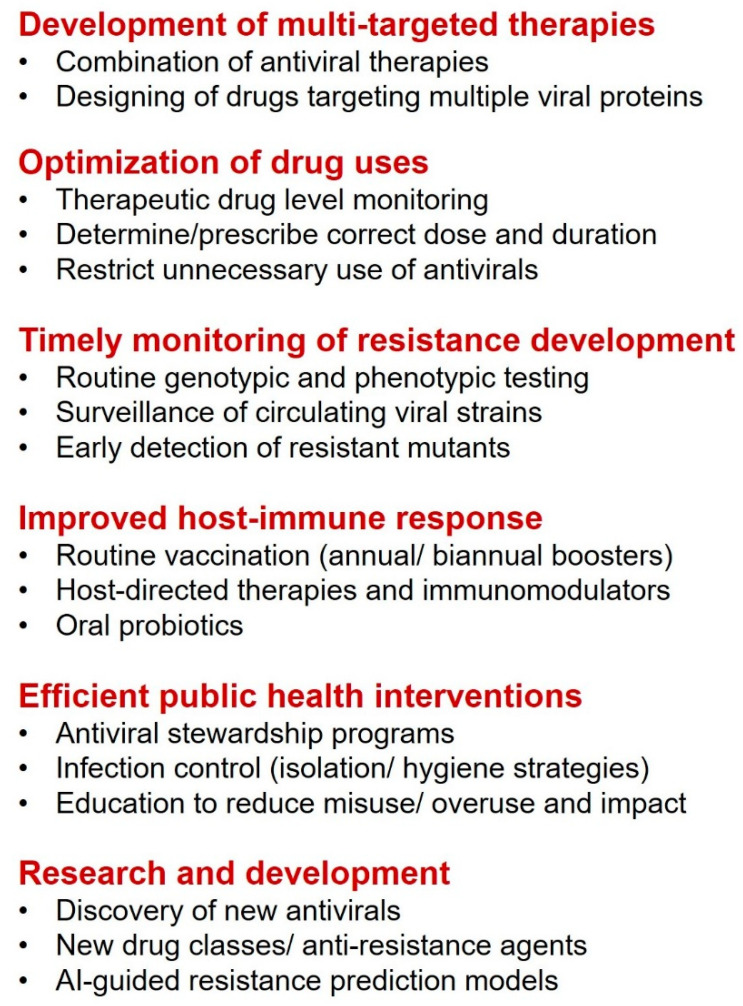
A flow diagram showing strategies to prevent antiviral drug resistance in SARS-CoV-2.

Currently, three approved antivirals (remdesivir, molnupiravir, nirmatrelvir) are available for the treatment of COVID-19. Both remdesivir and molnupiravir are nucleoside analogs that inhibit the viral replication by blocking RdRp, while nirmatrelvir disrupts the viral replication by directly inhibiting the SARS-CoV-2 Mpro. However, due to the emergence of specific mutations, resistance has been reported against all of the three approved key antivirals as well. To stop the spread of the possible emergence of antiviral resistance, proper management in clinical practices and treatment is needed along with rapid laboratory testing for proper surveillance (Figure 3). The selection of potent antivirals with less toxicity can combat the virus by using different antiviral mechanisms and also reducing the cross-resistance risk. Next, the use of next-generation sequencing in understanding the pattern or kinetics of antiviral drug-resistance patterns in the host population is another way to predict antiviral resistance [85]. Furthermore, genetic screening before starting the treatment for pre-existing resistance mutants for specific antiviral drugs may reduce the chances of resistance emergence and be helpful in the detection of new mutants [86]. Additionally, an authentic database of drug-resistance mutants should be developed to monitor the resistance pattern and to make subsequent decisions on therapeutic strategies [87] (Figure 3).

Next, immediate stoppage of the currently ongoing monotherapies while switching to the new antiviral therapies or adding another drug may help return the virus to its wild type that can then be easily eradicated with new drugs. Moreover, therapies using a combination of different drugs have always proven helpful in the treatment of antiviral-resistant mutants [88,89]. Notably, various combination therapies exhibit promising results against different mutants of SARS-CoV-2 and went through successful clinical trials while many of them have been approved and used in clinical settings as well [29,90]. Next, the transmission of antiviral drug-resistance mutants is the major problem after the emergence of new antiviral-resistance mutants. Prevention of transmission or blocking the spread of infection is another way to control the antiviral drug-resistance consequences. Transmission of viral infection could be controlled by simple and traditional measures like hand washing, proper sanitization, and vaccinations. Prevention of interspecies antiviral resistance virus transmission also plays an important role in antiviral drug-resistance management (Figure 3). In the case of coronaviruses, bats, rats, camels, and pangolins are known natural virus hosts, and the transmission of drug-resistant viruses to humans can be blocked by avoiding direct contact with them [91]. Effective wastewater treatment facilities may be further helpful in stopping the spread of the antiviral drugs to their natural hosts and thus the emergence of antiviral drug resistance [92]. Finally, rational drug design strategies such as enhanced interaction between viral targets and drugs by involving new binding cavities and allosteric sites, multivalent drugs, targeted covalent drugs, and resistant mutant-directed drug design might succeed in the battle against antiviral drug resistance in SARS-CoV-2 (Figure 3). Additionally, the discovery of new drug candidates with novel structures or mechanisms of action, targeted degradation of viral protein or RNA, and multi-target drugs including prior analysis of resistance development possibilities during the drug design would be potential strategies. Also, SARS-CoV-2 is reported to span well over the gut–lung axis, thus probiotics might be a potential option to fight against drug-resistant mutants of SARS-CoV-2 [93,94]. Above all, host-directed antiviral development and AI-based drug designing might provide alternative options to fight against and manage COVID-19 drug resistance [95,96] (Figure 3).

## 6. Future Perspectives

As the COVID-19 pandemic is ongoing, efforts for the development of new antivirals and therapeutic strategies are also ongoing worldwide. In the search to find out a potential antiviral or solution to combat SARS-CoV-2 infection, several drugs have been examined by using cell lines and animal models; however, their mechanism of action is not known experimentally. The testing approach without knowing the full mechanism of action does not yield positive hopes for the future; rather, it creates a selective pressure on the virus that leads to the emergence of drug resistance. Furthermore, if resistance is developed for the first generation of antivirals, it raises concerns for current approaches and prospects of antiviral resistance development. First, antiviral resistance arises when the virus continues to replicate in the presence of a drug, and subsequently, mutations appear to overcome the effect of drug presence. Second, the treatment-derived resistance that develops in the presence of high viral loads and mutability such as in the case of RNA viruses, but which is randomly determined, and successive resistance transmission are always followed by an outbreak. Additionally, SNPs play a great role in resistance development, and in the case of SARS-CoV-2, the nucleotide substitution frequency is ∼1  ×  10^−3^ substitutions per year [97]. Interestingly, spike protein mutations were shown to emerge only after three to four passages using Vero cells while SARS-CoV-2 was found to become fully adapted in animal models just within 6–10 serial infections and showed more infectivity and increased inflammatory response [98,99,100]. These adaptation studies provide a promising platform to examine the antiviral and vaccine efficacy that could be further used for drug-resistance development optimization.

Overall, time will tell the real scenario of antiviral resistance development in SARS-CoV-2 variants while there are many unanswered questions about the current ongoing COVID-19 pandemic, treatment strategies used, efforts made to mitigate the infection, and their future consequences. Therefore, surveillance of SARS-CoV-2-infected patients and sequencing of viral isolates is required to keep an eye on emerging mutations and subsequent antiviral drug resistance. Moreover, understanding the mechanism behind antiviral resistance to existing drugs given designing novel drugs to combat the antiviral resistance is necessary.

Key challenges and unresolved issues regarding SARS-CoV-2:

- Declining levels of neutralizing antibodies over time.
- Frequency of boosters and timing in different age groups.
- Development of new intranasal or oral vaccines.
- How do we control the rapid emergence of viral mutants?
- How do we design a broad-spectrum vaccine that protects current and future variants of SARS-CoV-2?
- What is the role of T-cell response in long-term protection?

## Figures and Tables

**Table 1:** Currently available FDA-approved antivirals.

Drug (Brand Name)	Class	Route of Administration	Mechanism of Actions	Limitations
Remdesivir (Veklury)	Nucleoside analog	Intravenous	Inhibits the RdRp of SARS-CoV-2	Specially reserved for severe COVID-19 conditions or individuals with a high risk of disease progression.
Nirmatrelvir + Ritonavir (Paxlovid)	3CL protease inhibitor	Oral	Inhibits the replication cycle of SARS-CoV-2	Not for patients under 12 years of age and also for mild to moderate COVID-19 conditions only.
Molnupiravir (Lagevrio)	Ribonucleoside analog	Oral	Inhibits the replication cycle of SARS-CoV-2	Not for patients under 18 years of age and also not indicated for severe COVID-19 or hospitalized patients.
Convalescent Plasma (OneBlood)	Anti-SARS-CoV-2 antibodies	Intravenous	Improves immunity against SARS-CoV-2	Only for immunocompromised or patients with immunosuppressive diseases.

**Table 2:** Key mutation of SARS-CoV-2 involved in the resistance development against antiviral drugs and strategies.

Associated Drug Target	Key Mutations	Affected Drug/Class	References
Nsp12 (RdRp)	E802D, S759A, V792I, C799F, V166L, F480L	Remdesivir/Nucleoside analog	[10,30,46,47,48]
Nsp5 (M pro)	S144M/F/A/G/Y, M165T, E166 V/G/A, H172Q/F, Q192T/S/L/A/I/P/H/V/W/C/F, T21I, P252L, T304I, F140L, L50F	Nirmatrelvir/3CL protease inhibitor	[32,33,49,50,51,52]
